# Altered expression of serum lncRNA CASC2 and miRNA-21-5p in COVID-19 patients

**DOI:** 10.1186/s40246-024-00578-9

**Published:** 2024-02-12

**Authors:** Shymaa E. Ayoub, Olfat G. Shaker, Mohamed Masoud, Essam A. Hassan, Eman M. Ezzat, Mona I. Ahmed, Randa I. Ahmed, Amal A. Ibrahim Amin, Fadwa Abd El Reheem, Abeer A. Khalefa, Rania H. Mahmoud

**Affiliations:** 1 Department of Medical Biochemistry and Molecular Biology, Faculty of Medicine, Fayoum University, Fayoum, 63514 Egypt; 2 Department of Medical Biochemistry and Molecular Biology, Faculty of Medicine, Cairo University, Cairo, Egypt; 3 Department of Public Health and Community Medicine, Faculty of Medicine, Fayoum University, Fayoum, Egypt; 4 Department of Tropical Medicine, Faculty of Medicine, Fayoum University, Fayoum, Egypt; 5 Department of Internal Medicine, Faculty of Medicine, Fayoum University, Fayoum, Egypt; 6 Department of Chest Disease and Tuberculosis, Faculty of Medicine, Fayoum University, Fayoum, Egypt; 7 Department of Medical Microbiology and Immunology, Faculty of Medicine, Fayoum University, Fayoum, Egypt; 8 Department of Clinical and Chemical Pathology, Faculty of Medicine, Fayoum University, Fayoum, Egypt; 9 Department of Physiology, Faculty of Medicine, Zagazig University, Zagazig, Egypt

**Keywords:** COVID-19, lncRNA CASC2, miRNA-21-p

## Abstract

Infection by severe acute respiratory syndrome coronavirus 2 (SARS-CoV-2) that causes coronavirus disease 2019 (COVID-19) has a high incidence of spread. On January 30, 2020, the World Health Organization proclaimed a public health emergency of worldwide concern. More than 6.9 million deaths and more than 768 million confirmed cases had been reported worldwide as of June 18, 2023. This study included 51 patients and 50 age- and sex-matched healthy subjects. The present study aimed to identify the expression levels of lncRNA CASC2 and miRNA-21-5p (also known as miRNA-21) in COVID-19 patients and their relation to the clinicopathological characteristics of the disease. The expression levels of noncoding RNAs were measured by RT-PCR technique. Results detected that CASC2 was significantly downregulated while miRNA-21-5p was significantly upregulated in COVID-19 patients compared to healthy subjects. A significant negative correlation was found between CASC2 and miRNA-21-5p. ROC curve analysis used to distinguish COVID-19 patients from controls. MiRNA-21-p serum expression level had a significant positive association with temperature and PO2 (*p* = 0.04 for each). These findings indicate that CASC2 and miRNA-21-p might be used as potential diagnostic and therapeutic biomarkers in COVID-19.

**Supplementary Information:**

The online version contains supplementary material available at 10.1186/s40246-024-00578-9.

## Introduction

The coronavirus disease 2019 (COVID-19) was originated in China in December 2019 as a result of the most recent human-infectious and pathogenic coronavirus which known as severe acute respiratory syndrome coronavirus 2 (SARS-CoV-2) [1, 2].

Following virus exposure, signaling cascades that result in the release of type I interferons, cytokines, and chemokines start the inflammatory response to COVID-19 [3]. Inflammasomes, multimeric protein complexes that are crucial for causing inflammation with the subsequent start of an adaptive immune response, are also activated by this initial exposure [4].

The effects of SARS-CoV-2 infection can range widely, from asymptomatic illness to potentially fatal lung disease combined with peripheral abnormalities [5]. Acute respiratory distress syndrome (ARDS) is a presenting symptom in severely affected patients who have lung injury, thromboembolic diseases, cardiovascular, cardiac, gastrointestinal dysregulation, and/or liver or kidney dysfunction [6].

LncRNAs are noncoding RNAs (ncRNAs) that are expressed endogenously and have a length greater than 200 nucleotides. LncRNAs act as regulatory molecules that mediate host–virus interactions [7]. Previous researches have demonstrated different lncRNAs that are connected to the occurrence and progression of COVID-19 disease [8–12].

LncRNA cancer susceptibility candidate 2 (CASC2) is located on chromosome 10q26. It functions as a tumor suppressor gene that can prevent cell growth, invasion, and metastasis while encouraging cell death in a variety of human cancers, including stomach cancer, papillary thyroid cancer, and pancreatic carcinoma [13]. Previous research has shown that lncRNA CASC2 prevents inflammation and sepsis-induced multi-organ damage through a variety of signaling pathways [14, 15].

MicroRNAs (miRNAs) are a family of tiny noncoding RNAs that range in length from 18 to 23 nucleotides. They regulate gene expression by attaching to a particular location in the 3′-untranslated region (3′-UTR) or open reading frame (ORF) to either degrade mRNA or prevent its translation [16]. Host-induced miRNAs can operate as pro- or antiviral factors, or they might assist the virus to evade immune response [17]. Studies have shown that SARS-CoV-2 enters host cells through penetration, and then, the virus releases its particles through exocytosis [18]. Additionally, microRNA plays a vital role in the development of both innate and adaptive immune cells by fine-tuning cell activities. Furthermore, host miRNAs have been reported to play a part in the cytokine storm linked to a SARS-CoV-2 infection [19]. A few of these miRNAs may be important regulators of mediators related to inflammation as well as inhibition of SARS-CoV-2 genome expression. [20–22].

MiRNA-21-5p (previously known as MiRNA-21) is a common miRNA that participates in numerous regulatory pathways and shows altered circulation levels in cancer and other illnesses, and the knowledge of its functions may help in developing new approaches to therapeutic intervention [23].

There is growing evidence suggesting that lncRNAs can function as competing endogenous RNAs (ceRNAs) by sponging miRNAs. They control the ability of miRNAs to prevent mRNAs from being translated into proteins [24]. Prior research has shown that in the colorectal cancer cell line [25] and the cervical cancer cell line [26], lncRNA CASC2 acts as a ceRNA to regulate miR-21. In our study, we revealed the expression profile of lncRNA CASC2 and miRNA-21-5p in cases with COVID-19 and explored their association with each other and with the clinicopathological manifestations of patients.

## Materials and method

### Subjects

Our study is a case control study conducted on 51 COVID-19 patients [29 males and 22 females]. A healthcare professional took nasopharyngeal samples and analyzed them for COVID-19 by real-time reverse transcription polymerase chain reaction (rRT-PCR). The rRT-PCR detection kits used for the patients in this study were manufactured by Certest Biotec. Co., Spain. Cases were chosen from the Internal Medicine and Chest departments of Egypt’s Fayoum University Hospital. Fifty healthy participants (30 males and 20 females) with negative COVID-19 PCR results made up the study’s negative control group. Before collecting samples, all participants gave their informed agreement, and the study obtained ethics committee approval from Fayoum University (R224).

*The CT quantitative assessment* was based on adding up the acutely affected areas in each of the five lung lobes, which were graded from 0 to 4 in the following ways:: 0 = (0%), 1 = (1–25%), 2 = (26–50%), 3 = (51–75%), or 4 = (76–100%). The overall score, which varies from 0 to 20, is the sum of the points from each lobe. Four categories of total severity scores (TSS) were analyzed: none (0), mild (1–5), moderate (6–10), and severe (11–20) [27].

*Chest X-Ray Scoring System (CXR score 18)*: the lungs were divided into six regions by two lines. Depending on the degree of the lung lesion, each location received a score ranging from 0 to 3. Score 0 for normal lung; 1 for interstitial infiltrates; 2 for interstitial and alveolar infiltrates combined (interstitial dominant); and 3 for alveolar and interstitial infiltrates combined (alveolar dominant). The six lung zones are scored, with scores ranging from 0 to 18 [28]. Then, patients are divided into 4 groups based on their overall CXR score, as follows: normal (group score of 0), mild (group score of 1–6), moderate (group score of 7–12), and severe (group score of 13–18) [29], by these new scoring system, COVID-19 patients’ disease severity can be determined.

### Routine tests

All patients underwent routine laboratory tests, such as complete blood counts (CBC), liver and kidney functions, blood gases, PH, S. Na, S. K, random blood glucose, and lactate dehydrogenase (LDH).

### RNA extraction

By utilizing a miRNeasy extraction kit (Qiagen, Valencia, CA, USA), total RNA was isolated from the serum. RNA's quantity and quality were assessed using the NanoDrop™ 2000 (Thermo Scientific, USA).

### Reverse transcription reactions

Following the manufacturer's instructions, RT2 first strand kit (Qiagen, Maryland, USA) was utilized for the synthesis of cDNA from the extracted RNA. In Addition, the miScript II RT Kit from Qiagen in Valencia, California, USA, was used to analyze the expression of miRNA in accordance with the protocol rules.

### Quantitative Real-time PCR

These reactions were achieved using RT2 SYBR Green PCR kit (Qiagen, Maryland, USA) for LncRNA CASC2 expression while miScript SYBR Green PCR kit (Qiagen, Valencia, CA, USA) for the detection of miRNA-21-5p.

The LncRNA CASC2 RefSeq Accession number was NR_026939. GAPDH served as an internal control for measuring CASC2 expression level [30].

Moreover, the miR-21-5p catalog number was MS0009079. SNORD 68 was used as an internal reference for miR-21-5p. After analysis of the data using the quantification of the cycle threshold (CT), relative expression of LncRNA CASC2 and miR-21-5p was calculated using the Eq. 2^−ΔΔCt^. Fold change (FC) values less than 1 indicate downregulation, while fold change values more than 1 indicate upregulation of noncoding RNAs [31, 32]. Control FC values were put as 1.

### Statistical analysis

The acquired data were statistically analyzed using SPSS software, version 22. For quantitative data, the mean, median, standard deviation (SD), and interquartile range (IQR) were calculated. Unpaired *t*-test was used to compare basic characteristics between the study groups. The Mann–Whitney-*U* test or the Kruskal–Wallis test was used to compare CASC2 and miRNA-21 (Log2) between the two groups or the three groups, respectively. The significance of qualitative data, which were presented as numbers and percentages, was assessed using the Chi-square [2] test. Spearman correlation was used to ascertain the association between the research parameters and LncRNA CASC2 and miRNA-21 (Log2). A ROC curve was used to identify the cutoff values for LncRNA CASC2 and miRNA-21 (Log2) as predictors in differentiating between cases and controls that had the maximum sensitivity and specificity. The cutoff for statistical significance was set at *P* < 0.05.

The sample size was calculated using (G power version 3.0.10). Minimal sample size of patients was at least 45 in each group assuming a power level of 0.80, alpha level of 0.05, and medium effect size of 0.6 between the two groups of the study for the study biomarkers.

## Results

### Clinical and demographic evaluation of the study groups

In this study, 50 people served as the controls, whereas 51 COVID-19-infected individuals were included. The average age of the patients was 60.63 ± 10.63 years, compared to 57.4 ± 7.39 years as the average age for the control group. Age and sex did not significantly differ between cases and controls (Table 1).

**Table 1:** Demographic and clinical characteristics

Characteristics	COVID-19 cases (*N* = 51)		Controls (*N* = 50)		*P*-value
Age (years)	60.63	10.63	57.4	7.39	0.079
Sex (*n*, %)					
Female	22	43.1%	20	40.0%	0.749
Male	29	56.9%	30	60.0%	
Systolic blood pressure (mmHg)	136.69	28.49			
Diastolic blood pressure (mmHg)	81.2	14.68			
Random blood sugar (mg/dL)	313.79	157.58			
Red blood cells (million/mm^3^)	4.34	0.73			
Hb% (g/dl)	11.85	2.34	13.18	1.34	0.001*
Mean cell volume (fl)	83.57	7.06	81.97	3.98	0.165
Mean cell hemoglobin (pg)	27.17	2.9	28.07	1.91	0.068
White blood cells	9.5	5.9			
Lymphocytes (%)	1.07	0.98			
Platelets count	222.4	83.8			
INR	1.42	0.5			
PT (seconds)	19.41	9.12			
CRP (mg/L)	44.43	30.49			
ALT (U/L)	82.62	120.1	30.18	6.68	0.003*
AST (U/L)	83.88	112.95	25.62	5.71	0.001*
Albumin (mg/dL)	2.97	0.49			
Serum creatinine (mg/dL)	1.77	1.95	0.76	0.17	0.001*
Lactate dehydrogenase (U/L)	432.49	210.15			

Data are represented as mean ± standard deviation or *n* (%). ^*^Significant at *P* < 0.05

**Fig. 1:**
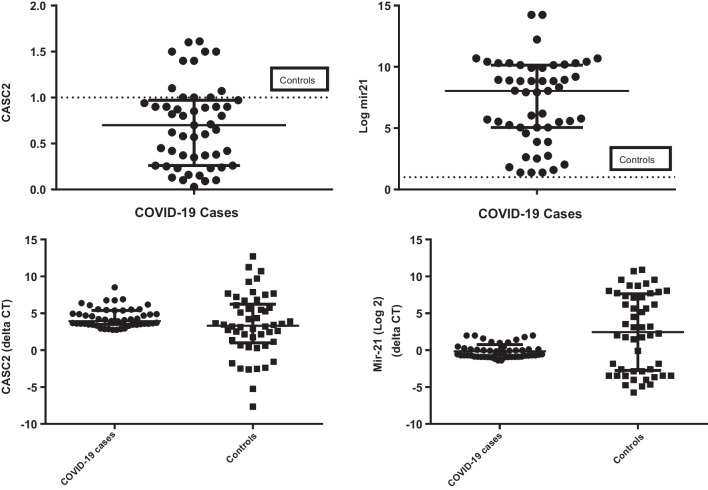
MiRNA-21 (log2) and lncRNA CASC2 expression levels in COVID-19 patients compared to healthy controls. Data are presented as dot plots. Fold change of expression levels of noncoding RNAs in the control group was set as 1. Data are expressed as median and intraquartile range

Regarding HB, ALT, AST, and creatinine, COVID-19 patients and the healthy group showed statistically significant differences (all *P* < 0.05). (Table 1). Table 2 identifies other clinical information about patients. Patients infected with the SARS-CoV-2 virus had median (IQR) serum expression levels of CASC2 of 0.7 (0.26–0.97) and miRNA-21 (log2) of 8.04 (5.06–10.15) (Fig. 1). When compared to controls, patients had significantly lower levels of LncRNA CASC2 and significantly higher levels of miRNA-21, both with a *p*-value < 0.001.

**Table 2:** Clinical and laboratory data of COVID-19 patients

Variables	COVID-19 cases (*N* = 51)	
GCS (mean ± SD)	14.11	2.51
Duration of admission (days) (mean ± SD)	9.2	5.56
*ICU admission (n, %)*		
No	18	35.3%
Yes	33	64.7%
*DM (n, %)*		
No	11	21.6%
Yes	40	78.4%
*Hypertension (n, %)*		
No	26	51.0%
Yes	25	49.0%
*Chronic heart disease (n, %)*		
No	48	94.1%
Yes	3	5.9%
*Chronic kidney disease (n, %)*		
CKD on HD	3	5.9%
No	48	94.1%
Temperature (mean ± SD)	37.26	0.68
Respiratory rate (mean ± SD)	29.39	7.89
Heart rate (mean ± SD)	91.82	24.72
PH (mean ± SD)	7.32	0.16
PCO2 (mean ± SD)	40.15	12.52
PO2 (mean ± SD)	54.4	23.88
HCO3 (mean ± SD)	20.59	6.11
S. Na (mean ± SD)	139.98	8.87
S. K (mean ± SD)	4.11	1.16
O2 on RA (mean ± SD)	83.82	7.56
O2 on oxygen (mean ± SD)	94.22	4.73
*Ventilation (n, %)*		
PEEP	11	21.6%
Mask	19	37.3%
CPAP	4	7.8%
Mechanical	17	33.3%
*GGO (n, %)*		
Yes	45	88.2%
No	6	11.8%
*Peripheral patches (n, %)*		
Yes	5	9.8%
No	46	90.2%
*Treatment (n, %)*		
*Ivermectin*		
No	46	90.2%
Yes	5	9.8%
*Remedesevir*		
No	36	70.6%
Yes	15	29.4%
*Favipiravir*		
No	50	98.0%
Yes	1	2.0%
*Ribavirin*		
No	50	98.0%
Yes	1	2.0%
*First PCR (n, %)*		
Negative	9	17.6%
Positive	42	82.4%
CO-RADS (mean ± SD)	4.54	0.73
*RSNA (n, %)*		
Atypical	1	2.0%
Undetermined	7	13.7%
Typical	42	82.4%
Percentage of GGO (mean ± SD)	46.87	17.45
Number of lobes affected (mean ± SD)	4.76	0.87
CXR score 18 (mean ± SD)	7.98	3.73
*CXR18 (n, %)*		
Mild (1–6)	20	39.2%
Moderate (7–12)	25	49.0%
Severe (13–18)	6	11.8%
TSS total score (mean ± SD)	8.68	4.01
TSS total score (0–20) (*n*, %)		
None (0)	1	1.9%
Mild (1–5)	21	41.2%
Moderate (6–10)	29	56.9%

GCS, Glasgow Coma Scale; HD, hemodialysis; O2 on RA, oxygen on room air; PEEP, positive end-expiratory pressure; CPAP, continuous positive airway pressure; GGO, ground-glass opacification/opacity; CO-RADS (COVID-19) Reporting and Data System; RSNA, Radiological Society of North America Chest CT Classification System; CXR score 18, Modified Chest X-Ray Scoring System in Evaluating Severity of COVID-19 (range from 0 to 18); TSS, total severity score (range from 0 to 20). Data are represented as mean ± standard deviation or n (%)

### Relation of the serum levels of LncRNA CASC2 and miR-21-5p with demographic, clinical, and laboratory variables of COVID-19 patients

Table 3 shows that PEEP (positive end-expiratory pressure) versus CPAP (continuous positive airway pressure), and CPAP versus mechanical ventilation were significantly associated with miRNA-21-5p serum expression level,* p* = 0.01. Furthermore, CXR18 (Chest X-Ray) Scoring System was significantly related to the expression level of miRNA-21-5p, *p* = 0.009.

**Table 3:** Relation between clinical data and biomarkers

	CASC2			*P*-value	MiRNA-21 (log2)			*P*-value
	Median	IQR			Median	IQR		
*Sex*								
Female	0.67	0.23	0.94	0.676	8.45	5.5	10.15	0.621
Male	0.7	0.37	1		7.93	3.88	9.92	
*ICU admission*								
No	0.85	0.58	1	0.214	8.93	5.71	10.15	0.265
Yes	0.57	0.26	0.9		7.93	4.58	9.19	
*DM*								
No	0.85	0.57	1	0.303	7.93	5.06	8.84	0.551
Yes	0.64	0.25	0.96		8.2	4.82	10.17	
*Hypertension*								
No	0.84	0.38	1.07	0.073	7.06	2.74	9.92	0.246
Yes	0.6	0.23	0.89		8.06	5.5	10.3	
*CHD*								
No	0.68	0.26	0.96	0.691	7.93	4.82	10.03	0.268
Yes	0.85	0.35	1.1		8.84	8.04	14.24	
*CKD*								
CKD on HD	0.65	0.26	0.8	0.663	2.63	1.82	9.19	0.268
No	0.71	0.3	0.99		8.05	5.06	10.15	
*Ventilation*								
1 (PEEP)	0.6	0.13	0.97	0.197	8.84	6.03	10.15	0.010* 1 vs 3 3 vs 4
2 (mask)	0.82	0.37	0.94		5.71	5.06	9.92	
3 (CPAP)	1.45	0.83	1.56		1.71	1.38	2.33	
4 (mechanical)	0.45	0.35	0.9		8.84	5.78	10.42	
*GGO*								
Yes	0.71	0.31	0.96	0.691	7.93	4.82	10.03	0.251
No	0.6	0.1	1		9.92	8.06	10.3	
*Peripheral patches*								
Yes	0.89	0.6	1	0.701	8.95	8.06	9.92	0.284
No	0.68	0.26	0.94		7.93	4.58	10.15	
*Ivermectin*								
No	0.68	0.35	0.97	0.468	7.93	4.58	10.15	0.206
Yes	0.7	0.15	0.8		8.9	8.84	9.92	
*Remedesevir*								
No	0.8	0.32	1	0.222	7.06	4.23	10.03	0.368
Yes	0.58	0.15	0.85		8.84	5.5	10.15	
*Favipiravir*								
No	0.71	0.35	0.97	0.078	7.99	5.06	9.92	0.118
Yes	0.09	0.09	0.09		12.23	12.23	12.23	
*Ribavirin*								
No	0.71	0.35	0.97	0.196	8.05	5.06	10.15	0.667
Yes	0.13	0.13	0.13		5.5	5.5	5.5	
*First PCR*								
Negative	0.38	0.23	1	0.443	8.84	6.2	9.92	0.400
Positive	0.71	0.37	0.94		7.93	3.88	10.15	
*RSNA*								
Atypical	0.24	0.24	0.24	0.506	5.71	5.71	5.71	0.621
Undetermined	0.26	0.23	1		5.58	2.63	9.92	
Typical	0.71	0.37	0.9		8.59	5.06	10.15	
*CXR18 (0–18)*								
Mild	0.76	0.4	0.95	0.342	5.52	3.31	8.2	0.009* Mild vs Severe
Moderate	0.7	0.35	1.07		8.84	5.26	10.15	
Severe	0.45	0.23	0.8		10.56	9.19	10.69	
*TSS total score (0–20)*								
None	0.24	0.24	0.24	0.462	5.71	5.71	5.71	0.540
Mild	0.80	0.37	1.00		6.20	4.58	9.92	
Moderate	0.65	0.35	0.94		8.84	5.06	10.15	

### Correlation of LncRNA CASC2 and miRNA-21-5p serum expression levels with characteristics of COVID-19 patients

LncRNA CASC2 and miRNA-21-5p serum expression levels had a significant negative association with each other (*p* = 0.012). miRNA-21-5p level had a significant positive correlation with temperature and PO2 (*p* = 0.04 for each) (Table 4).

**Table 4:** Correlation between the expression levels of lncRNA CASC2 and miRNA- 21and clinical and laboratory data of COVID-19 patients

	CASC2	MiRNA−21 (log2)
*Log2 of miRNA-21*		
*r*	− .351*	
*P*	0.012	
*Age*		
*r*	− 0.062	0.205
*P*	0.668	0.149
*SBP*		
*r*	− 0.172	0.254
*P*	0.229	0.072
*DBP*		
*r*	− 0.042	0.157
*P*	0.768	0.271
*GCS*		
*r*	0.233	− 0.024
*P*	0.115	0.875
*Duration of admission*		
*r*	0.099	− 0.127
*P*	0.489	0.373
*Temperature*		
*r*	− 0.078	.291*
*P*	0.592	0.04
*Respiratory rate*		
*r*	0.003	− 0.034
*P*	0.984	0.815
*HR*		
*r*	− 0.139	− 0.016
*P*	0.331	0.912
*PH*		
*r*	0.106	0.22
*P*	0.461	0.121
*PCO2*		
*r*	0.005	− 0.054
*P*	0.971	0.706
*PO2*		
*r*	− 0.046	.277*
*P*	0.749	0.049
*HCO3*		
*R*	− 0.029	0.161
*P*	0.842	0.264
*S. Na*		
*r*	− 0.068	− 0.061
*P*	0.633	0.673
*S. K*		
*r*	− 0.015	− 0.173
*P*	0.919	0.225
*O2 on RA*		
*r*	0.247	− 0.028
*P*	0.08	0.843
*O2 on oxygen*		
*r*	0.151	− 0.099
*P*	0.289	0.489
*RBS*		
*r*	− 0.074	0.045
*P*	0.605	0.755
*RBCs*		
*r*	0.129	0.107
*P*	0.369	0.456
*Hg (g/dl)*		
*r*	0.136	0.01
*P*	0.342	0.947
*MCV (fl)*		
*r*	− 0.005	− 0.142
*P*	0.973	0.322
*MCH (pg)*		
*r*	− 0.007	− 0.035
*P*	0.962	0.808
*Lymphocyte*		
*r*	0.147	− 0.065
*P*	0.302	0.649
*INR*		
*r*	0.108	− 0.252
*P*	0.455	0.077
*PT*		
*r*	0.112	− 0.258
*P*	0.434	0.068
*CRP*		
*r*	− 0.002	0.232
*P*	0.991	0.101
*ALT*		
*r*	0.081	− 0.087
*P*	0.572	0.545
*AST*		
*r*	− 0.057	− 0.031
*P*	0.692	0.827
*S. Creatinine*		
*r*	− 0.161	− 0.165
*P*	0.258	0.246
*LDH*		
*r*	0.115	− 0.027
*P*	0.422	0.848
*CO-RADS*		
*r*	0.042	0.241
*P*	0.77	0.091
*TSS total score*		
*r*	− 0.019	0.126
*P*	0.898	0.381
*Percentage of GGO*		
*r*	− 0.055	0.047
*P*	0.705	0.745
*Number of lobes affected*		
*r*	0.146	− 0.033
*P*	0.313	0.821

MiRNA-21-5p level had a significant positive correlation with temperature and PO2 (*p* = 0.04 for each)

Diagnostic performance of CASC2 and miRNA-21-5p in COVID-19.

**Fig. 2:**
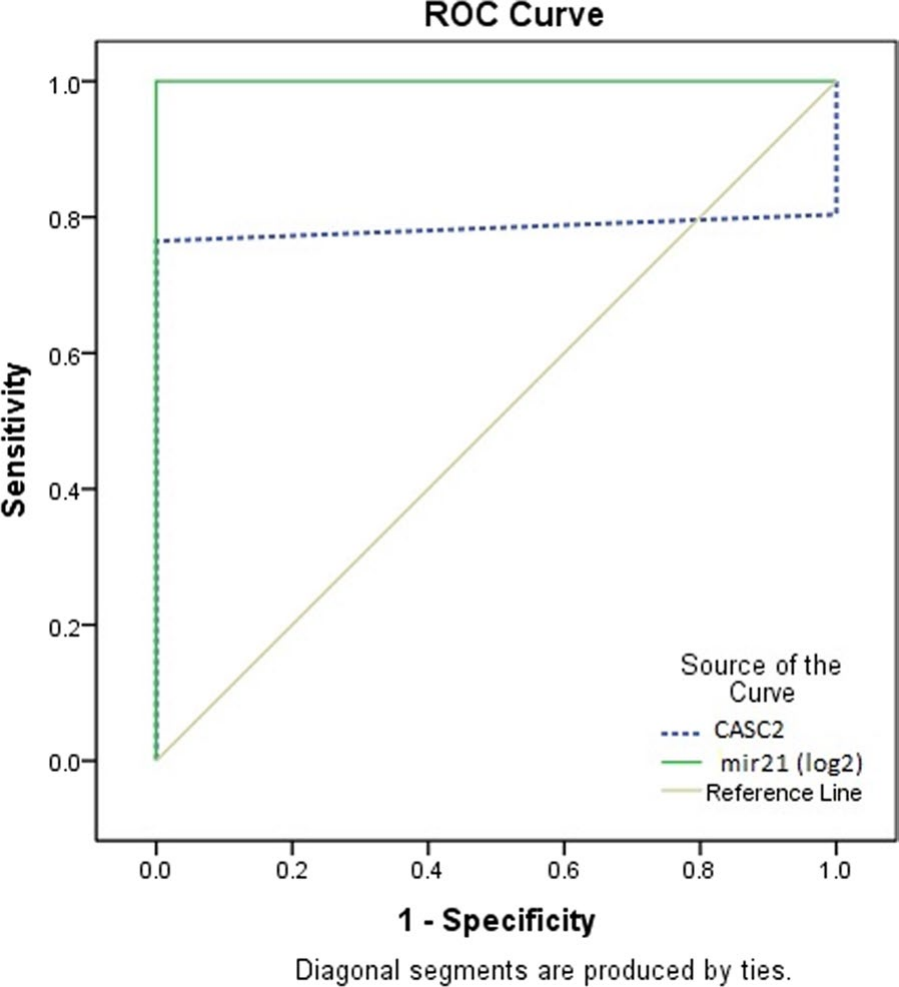
Receiver operating characteristic (ROC) curve analysis of serum miRNA-21 and lncRNA CASC2 was used to distinguish between COVID-19 patients and the control group. AUC, the area under the curve

ROC curve showed that CASC2 can discriminate COVID-19 patients from healthy people (AUC = 0.784, 95% CI (0.674–0.895), *p* < 0.001) with a sensitivity of 76.5% and a specificity of 100% at a cutoff > 0.987 (fold). Serum miRNA-21-5p also identify COVID-19 patients from healthy controls (AUC = 1.00, 95% CI (1–1), *p* < 0.001) with a sensitivity and specificity of 100% for each of them at a cutoff point 1.19 (fold) (Table 5, Fig. 2).

**Table 5:** Receiver operating characteristics curve (ROC) analysis using serum lncRNA CASC2 and miRNA-21 for differentiating COVID-19 patients from the control group

	AUC (95%CI)	*P*-value	Cutoff point	Sensitivity (%)	Specificity (%)
lncRNA CASC2	0.784 (0.674–0.895)	< 0.001*	0.987	76.5	100.0
miRNA-21 (log2)	1.000 (1.000–1.000)	< 0.001*	1.19	100.0	100.0

AUC, the area under the curve; CI, confidence interval. *Significant at *P* < 0.05

## Discussion

In COVID-19 disease, LncRNAs have been implicated in a growing number of biological regulatory processes, including immune disorders, thrombosis, and a severe inflammatory response [33–35]. But these lncRNAs’ features and how they work in COVID-19 remain unclear [36].

Previous research have shown that the long noncoding RNA (lncRNA) cancer susceptibility candidate gene 2 (CASC2), which is found on chromosome 10 of the human genome, inhibits inflammation and sepsis-induced multi-organ damage through a variety of signaling pathways, and inflammation caused by the NF-kB signaling pathway was observed to be inhibited by the lncRNA CASC2 in human renal tubular epithelial cells [37]. Similar to the previous study, it was found that miR-545-3p/PPAR axis modulation via the overexpression of lncRNA CASC2 protects against acute lung injury and damage to human or embryonic kidney cells caused by lipopolysaccharides [38, 39]. Based on the aforementioned supporting data, the lncRNA CASC2 was thought to have potential as a biomarker for inflammation control. Nevertheless, few investigations have shown that. Consequently, the purpose of this study aimed to investigate for the first time the involvement of lncRNA CASC2 in COVID-19. We reported that lncRNA CASC2 was significantly downregulated in COVID-19 patients than in healthy controls.

LncRNAs may behave as ceRNAs that influence the concentration and biological activity of miRNAs, according to previous studies [40, 41]. The function of CASC2 as a ceRNA of miRNA-21 was identified via bioinformatics analysis. It has been established that lncRNA CASC2 is one of miRNA-21’s primary target genes. In a sequence-specific manner, miRNA-21 was able to reduce the expression of CASC2 [42, 43].

When compared to healthy participants, miRNA-21-5p expression levels were markedly increased in COVID-19 patients. Furthermore, a strikingly negative correlation between CASC2 and miRNA-21-5p expression levels in the serum of COVID-19 patients was observed.

The authors revealed increased levels of miRNA-21 in acute COVID-19-infected individuals when compared with patients had Influenza-acute respiratory distress syndrome and healthy subjects [23]. In addition, a positive association was detected between the expression level of miRNA-21 and the number of intensive care unit (ICU) days on extracorporeal membrane oxygenation or ventilation and dialysis. In accordance with a study done by Dingsdag et al. we detected a negative correlation but without significance between miRNA-21 expression level and levels of lactate dehydrogenase enzyme (LDH) [44]. Interestingly, the upregulation of fibrosis associated miRNA-21 in COVID-19 survivors might be a predictor of inflammation and chronic myocardial damage [45].

It was determined that miRNA-21 has binding sites on coronaviruses. Thus, as a result of SARS-CoV-2 infection, the expression levels of miRNA-21 in blood may change [46]. Thus, understanding its behavior could help to develop new approaches to therapeutic interventions [23, 47].

As a result of SARS-CoV-2 lung infection, Nersisyan et al. found that miR-21-3p is eightfold upregulated. According to reports, SARS-CoV-2 properly interacts with host miR-21-3p in the early stages of infection to block the host’s immune response by directly binding to the viral genome, delaying the immune response and enhancing viral survival and reproduction [48]. IL-17, a proinflammatory cytokine involved in the pathophysiology of various autoimmune disorders, can be increased by miRNA-21 [49].

Notably, the expression of miRNA-21 may provide promising SARS-CoV-2 infection diagnostic value. Additionally, because of the presence of antiviral miRNAs and antibodies, it has been shown that patients with COVID-19 can be treated using plasma from persons who have recovered from SARS-CoV-2 infection. Additionally, nasal spray or drops can be utilized to provide nano-based miRNA vaccinations. Due to the respiratory system being the frequent initial site for SARS-CoV-2 viral entry, the nasal spray variant of the nano-vaccine appears to be more effective against COVID-19 illness [50].

Regarding the clinical data, 21.6% of the patients was on PEEP, 37.3% on mask, 7.8% on CPAP, 33.3% on mechanical ventilation, 45% of the patients have ground-glass opacification (GGO). A cytokine storm that damages alveolar structures due to dysregulated immune systems can facilitate the virus’s entry into vascular endothelial cells through the blood–air barrier. Endothelial dysfunction increases the pulmonary arteries' rigidity and vulnerability as the disease progresses, which eventually leads to thrombosis and microvessel obstruction in alveolar capillaries, which may result in hypoxemia or pulmonary hypertension [51].

Multifocal ground-glass opacity (GGO) with rounded morphology with characteristic bilateral peripheral distribution is the classic chest CT result for COVID-19 pneumonia. This finding can be linked to consolidation and crazy-paving patterns [52]. In addition, traction bronchiectasis and vascular dilatation are common GGO findings in COVID-19 patients [53].

## Conclusion

We demonstrated for the first time that lncRNA CASC2 is downregulated in the serum of COVID-19 patients, which is probably protective against SARS-CoV-2 infection. According to our research, patients with COVID-19 may benefit from the therapeutic use of the lncRNA CASC2. Despite the relatively limited number of patients included in this research, our study provides a starting point for more extensive research which should be used to examine the long-term prognosis of COVID-19 patients.

### Supplementary Information

**Additional file 1**. Raw expression data of lncRNA CASC2 and miRNA-21-5p.

## Data Availability

All relevant data are included in the article.
