# Supplementary material for: Altered expression of serum lncRNA CASC2 and miRNA-21-5p in COVID-19 patients
Source: Hum Genomics. 2024 Feb 12;18:18. doi: 10.1186/s40246-024-00578-9 (PMC10860220; doi:10.1186/s40246-024-00578-9)
Supplement: Supplementary file 1 — Additional file 1. Raw expression data of lncRNA CASC2 and miRNA-21-5p. [file 40246_2024_578_MOESM1_ESM.docx]

Raw expression data of lncRNA CASC2 and miRNA-21-5p

| miRNA-21-5p | | |  | lncRNA CASC2 | |
| --- | --- | --- | --- | --- | --- |
| Patients delta CT | Control delta ct |  | delta ct patients (mean of Control) | delta ct patients | delta ct control |
| -0.694 | -5.74 |  | 6.179 | 5.48 | 2.743 |
| -1.162 | -1.84 |  | 6.916 | 4.13 | 0.656 |
| -0.704 | -3.44 |  | 6.764 | 6.23 | 2.908 |
| -0.142 | 2.02 |  | 3.957 | 1.1 | 0.585 |
| -0.009 | -4.04 |  | 6.385 | -2.29 | -5.233 |
| -0.893 | -3.5 |  | 4.228 | -0.78 | -1.566 |
| -0.712 | -3.44 |  | 4.132 | -1.92 | -2.610 |
| -0.915 | -2.86 |  | 4.179 | -1.8 | -2.537 |
| -0.694 | 3.12 |  | 2.857 | 3.75 | 4.335 |
| 0.494 | -2.6 |  | 3.486 | -1.73 | -1.774 |
| 1.117 | 2.26 |  | 3.643 | 3.66 | 3.459 |
| 1.429 | 1.74 |  | 2.957 | 3.14 | 3.625 |
| 0.055 | 1.47 |  | 4.876 | 2.56 | 1.126 |
| 1.781 | 4.5 |  | 2.957 | 3.74 | 4.225 |
| -0.182 | 3.56 |  | 3.442 | 5.25 | 5.250 |
| -0.900 | -0.09 |  | 3.594 | 1.53 | 1.378 |
| -0.063 | 3.19 |  | 5.501 | 5.28 | 3.221 |
| -0.893 | 7.18 |  | 3.531 | 5.21 | 5.121 |
| -0.712 | 5.66 |  | 3.610 | 5.79 | 5.622 |
| -0.915 | 9.56 |  | 5.442 | 9.68 | 7.680 |
| 0.996 | 10.91 |  | 2.764 | 12.04 | 12.718 |
| 0.494 | 8.04 |  | 4.594 | 9.03 | 7.878 |
| 1.055 | 5.18 |  | 5.385 | 7.41 | 5.467 |
| -0.860 | 7.73 |  | 3.344 | 3.12 | 3.218 |
| -1.382 | -4.91 |  | 8.501 | -2.59 | -7.649 |
| -0.537 | 6.19 |  | 3.594 | 7.82 | 7.668 |
| -1.382 | 7.71 |  | 3.304 | 9.58 | 9.718 |
| 0.255 | 8.05 |  | 4.694 | 8.48 | 7.228 |
| -0.915 | 10.72 |  | 5.562 | 11.39 | 9.270 |
| -0.694 | -4.74 |  | 4.838 | 5.29 | 3.894 |
| -0.931 | -1.84 |  | 4.694 | 3.78 | 2.528 |
| -0.750 | -3.44 |  | 4.063 | -1.77 | -2.391 |
| -0.017 | 2.02 |  | 3.594 | -2.33 | -2.482 |
| 1.586 | -4.04 |  | 3.764 | 3.75 | 3.428 |
| -0.860 | -3.66 |  | 3.442 | 5.73 | 5.730 |
| 0.111 | -3.44 |  | 3.936 | 2.66 | 2.166 |
| 1.985 | -2.86 |  | 2.857 | 6.14 | 6.725 |
| -0.030 | 3.12 |  | 5.385 | 4.56 | 2.617 |
| -0.557 | -2.6 |  | 3.676 | 7.74 | 7.506 |
| -0.694 | 5.66 |  | 4.957 | 3.25 | 1.735 |
| -0.931 | 9.56 |  | 5.562 | 2.53 | 0.410 |
| -0.081 | 7.91 |  | 4.876 | 8.28 | 6.846 |
| -0.608 | 9.04 |  | 6.086 | 6.21 | 3.566 |
| 1.985 | 7.13 |  | 2.755 | 1.79 | 2.477 |
| 1.985 | 8.73 |  | 2.857 | 10.68 | 11.265 |
| -0.968 | -4.66 |  | 3.764 | 11.04 | 10.718 |
| -0.561 | 6.19 |  | 6.764 | 5.48 | 2.158 |
| 0.111 | 7.35 |  | 3.442 | 3.13 | 3.130 |
| -0.537 | 8.75 |  | 3.594 | 6.23 | 6.078 |
| 0.111 | 1.75 |  | 4.253 | 1.1 | 0.289 |
